# A Unique Case of Concomitant T‐Prolymphocytic Leukemia and B‐Cell Acute Lymphoblastic Leukemia

**DOI:** 10.1002/jha2.70252

**Published:** 2026-02-18

**Authors:** Viral M. Patel, Jonathan Hyak, Soolmaz Laknezhad, Monica Chintapenta, Prapti Patel, Weina Chen, Radhika Kainthla

**Affiliations:** ^1^ Department of Internal Medicine Division of Hematology/Oncology University of Texas Southwestern Dallas Texas USA; ^2^ Parkland Health and Hospital System Dallas Texas USA; ^3^ Department of Pathology University of Texas Southwestern Dallas Texas USA; ^4^ Department of Pharmacy Parkland Health Dallas Texas USA

**Keywords:** alemtuzumab, B‐cell acute lymphoblastic leukemia, *JAK3*, *KMT2A*, *TCL1*, T‐prolymphocytic leukemia

## Abstract

T‐prolymphocytic leukemia (T‐PLL) is a rare lymphoid malignancy with a poor prognosis. B‐cell acute lymphoblastic leukemia (B‐ALL) also confers a poor prognosis, especially in patients with high‐risk features without an option for transplant. Here, we present a case of a patient with T‐PLL initially treated with multi‐agent chemotherapy who then developed B‐ALL, the management strategies, and possible pathogenesis of two concurrent rare malignancies. One proposed mechanism for the development of both hematologic malignancies in this patient is the acquisition of a *KMT2A* rearrangement, raising the possibility of clonal evolution resulting in therapy‐related or secondary leukemia. Another explanation is the presence of a common clonal stem cell progenitor harboring a *JAK3* mutation.

## 1 Introduction

T‐prolymphocytic leukemia (T‐PLL) is a rare and aggressive lymphoid malignancy that carries a poor prognosis with a 5‐year overall survival of 21% [1]. Aberrancies in the oncogenes T‐cell leukemia‐1 (*TCL1*) and ataxia telangiectasia‐mutated gene (*ATM*) have been implicated in the development of T‐PLL, whereby a mutated TCL1 shortens telomeres and induces apoptosis, and a mutated ATM fails to correct DNA double‐stranded breaks [2]. Mutations in both genes lead to leukemogenesis, and the acquisition of subsequent alterations in the Janus Kinase 3 (*JAK3*) gene leads to further tumorigenesis [2]. When indicated, first‐line treatment is intravenous alemtuzumab, a monoclonal antibody that targets CD52, a marker highly expressed in T‐PLL. In one study, alemtuzumab resulted in 60% complete response rate and 16% partial response rate [3]. Pentostatin may be added to alemtuzumab in T‐PLL that is slow to respond, and stem cell transplant is considered to maintain remission [4, 5].

B‐cell acute lymphoblastic leukemia (B‐ALL) is another aggressive blood cancer that has a bimodal distribution, with 80% of cases occurring in children and a second peak at 50 years of age [6]. Treatment has traditionally involved multi‐agent chemotherapy followed by maintenance therapy or allogeneic hematopoietic stem cell transplant for high‐risk disease [7]. Therapy‐related/secondary B‐ALL (t‐BALL) is uncommon and accounts for 2%–10% of all B‐ALL [8]. T‐BALL has particular molecular patterns, including a higher frequency of *KMT2A* rearrangement and myelodysplastic syndrome‐like chromosomal abnormalities. In one report, 63% of 116 patients with t‐BALL had prior anthracycline exposure, 33% had prior Topoisomerase II inhibitors, and 30% received both. In addition, 13% had a *KMT2A* gene rearrangement. Patients who had received prior Topoisomerase II inhibitors had a median latency of 4.7 years (range: 0.5–16 years), and those who had a *KMT2A* rearrangement had a median latency of 2 years (range: 1–6 years). Thirty percent of patients with t‐BALL with a *KMT2A* rearrangement had received a topoisomerase II inhibitor in this report [9].

Here, we present a patient who developed T‐PLL followed by B‐ALL, review the management strategy, and hypothesize about the pathogenesis of these two rare malignancies co‐occurring in the same patient.

## 2 Case Presentation

**FIGURE 1:**
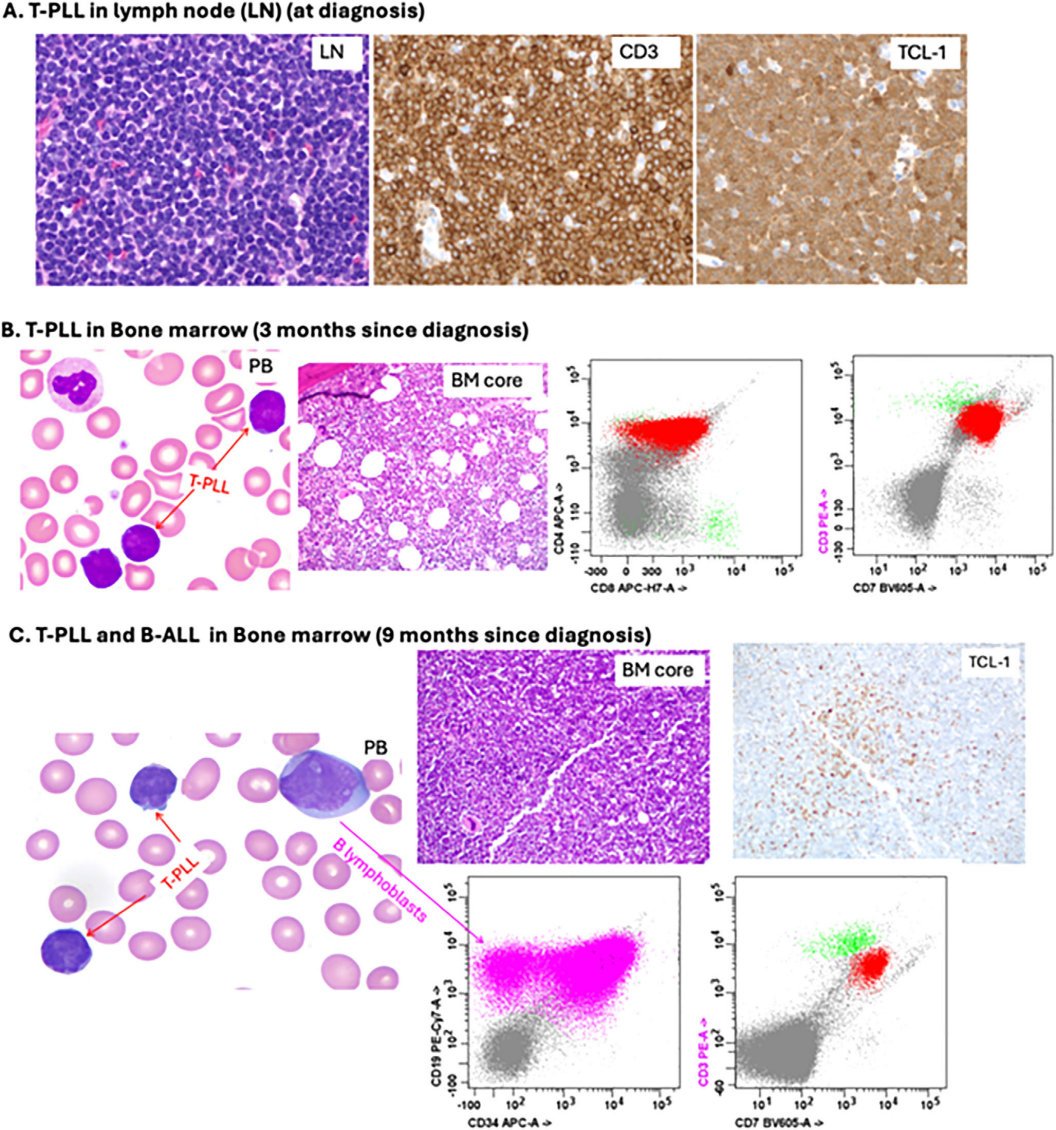
T‐prolymphocytic leukemia (T‐PLL) and B‐acute lymphoblastic leukemia (B‐ALL) by morphology and immunophenotype. (A) T‐PLL in lymph node (LN) at diagnosis: LN demonstrating infiltration by small to medium‐sized lymphoma cells that are positive for CD3 and TCL‐1 by immunohistochemistry (IHC). (B) T‐PLL in bone marrow (BM) at 3 months since diagnosis: Scattered small to medium‐sized lymphoma cells with irregular nuclei and scant cytoplasm in peripheral blood (PB) and BM. Lymphoma cells (in red) are positive for CD3(dim), CD4, CD8(partial), and CD7 by flow cytometry (FC). Normal T cells are in green. (C) T‐PLL and B‐ALL in bone marrow (9 months since diagnosis): Scattered small to medium‐sized lymphoma cells and medium‐sized to large B lymphoblasts with fine chromatin and distinct nucleoli in PB and BM. T‐PLL is positive for TCL‐1 (IHC) and CD3(dim)/CD7 (FC), while B‐ALL is positive for CD19 and CD34 (most) by FC (in violet).

A 51‐year‐old woman presented with subjective fevers and lethargy and was noted to have a white blood cell (WBC) count of 54 × 10^9^/L, hemoglobin (Hb) 13.1 g/dL, and platelets (Plt) 244 × 10^9^/L. Her differential showed 80% lymphocytes, 14% neutrophils, and 3% “atypical” cells. Computed tomography (CT) of the neck, chest, abdomen, and pelvis with contrast showed enlarged axillary lymph nodes and subpectoral lymph nodes of 1.9 centimeters (cm), right hilar lymph nodes of 1.7 cm, and multiple cervical lymph nodes, largest 0.9 cm. She underwent excisional lymph node biopsy of a submental lymph node showing a CD3(+)/CD4(+) T‐cell lymphoma with CD30(‐)/TCL1(+) by immunohistochemical staining (Figure 1A). A peripheral blood (PB) flow cytometry showed a 69% population of aberrant CD3(+)/CD4(+)/CD7(+)/CD8(+) T‐lineage cells. Cytogenetic analysis revealed a normal female karyotype in all 18 cells examined (46, XX). The overall findings were consistent with peripheral T‐cell lymphoma (PTCL). A baseline PET/CT scan showed adenopathy above and below the diaphragm as well as activity in bone marrow (BM).

Due to suspected Stage IV PTCL, she started treatment with CHOEP—a combination of cyclophosphamide, doxorubicin, vincristine, etoposide, and prednisone. After three cycles of CHOEP, although her constitutional symptoms disappeared, her WBC count was unimproved at 59 × 10^9^/L, and a PET/CT was without any change in avidity in lymph nodes with persistent activity in BM. Due to concern for refractory disease, the pathology was re‐evaluated on initial submental lymph node biopsy, which demonstrated TCL1 expression. Thereafter, a *TCL1* FISH on PB was collected and was positive in 169 of 200 (84%) of the cells examined (probe provided by CytoCell). ICSN: nuc ish(TCL1Ax2) (3'TCL1A sep 5'TCL1Ax1)[169/200]. A BM biopsy showed 60%–70% involvement of T‐PLL with a similar immunophenotype in PB and BM (Figure 1B). Karyotype of the bone marrow sample revealed multiple abnormalities in 10 of 42 cells analyzed: copy loss of chromosomes 6, 10, 11, 14, 21, and 22; deletion of the terminal end of the long arm of Chromosome 6; isochromosome 8q resulting in gain of 8q and loss of 8p; and interstitial deletion within the short arm of Chromosome 1. In addition, there was genetic material of uncertain origin found on several chromosomes, as well as 1–2 marker chromosomes. FoundationOne next‐generation sequencing (NGS) from the BM had alterations in *ATM* K2811del (variant allele frequency [VAF] 61.8%) and *JAK3* M511I (VAF 39.7%). A *TP53* mutation was not identified among the several hundred genes assessed via this assay. Human T‐lymphotropic Virus 1 antibodies were negative. A definitive diagnosis of T‐PLL was made.

Because the patient was feeling very well and without symptoms, she was on observation for four additional months, until she was noted to have new neutropenia and thrombocytopenia (WBC 161 × 10^9^/L, undetectable neutrophils with 100% lymphocytes, Hb 11.3 g/dL, Plt 66 × 10^9^/L). She began first‐line treatment for T‐PLL with intravenous alemtuzumab three times a week. Weekly pentostatin was added at Week 5 as she had persistent leukocytosis concerning for refractory disease (WBC 150 × 10^9^/L with undetectable neutrophils and 100% lymphocytes, Hb 8.5 g/dL, Plt 66 × 10^9^/L) [4]. After 7 weeks of alemtuzumab and 3 weeks of pentostatin, she developed severe cytopenias (WBC 44 × 10^9^/L with undetectable neutrophils and 57% lymphocytes, Hb 5.3 g/dL, Plt < 5 × 10^9^/L). As a result, a PB flow cytometry was conducted to assess her disease burden. It showed a 47% population of B‐lymphoblasts and a 48% population of T‐lineage cells, indicating concomitant B‐ALL and T‐PLL. B‐lymphoblasts were CD10(‐)/CD19(+)/CD34(most +)/CD20(‐) by flow cytometry. A BM biopsy confirmed this result, with 100% cellularity, with 90% involvement of B‐ALL and 10% T‐PLL (Figure 1C). Full cytogenetics could not be completed due to the limited specimen. NGS from PB (due to dry tap) revealed a *KMT2A (MLL)::MLLT1* fusion, *JAK3* M511I (VAF 41.3%), *RB1* rearrangement exon 4, and *ATM* K2811del (70.2%); no *TP53* mutation was identified. A PB FISH panel did not detect a *BCR::ABL1* fusion but did show 8q24.2 (*MYC*) gain, 9q34 (*ABL/ASS1*) loss, 11q23 (*KMT2A*) loss, 11q23 (*KMT2A*) rearrangement, and 22q11.2 (*BCR*) loss. The diagnosis of B‐ALL was made about 8 months from the start of the first cycle of CHOEP, and about 6 months after the start of the final cycle of CHOEP.

In hopes of targeting both her B‐ALL and T‐PLL, she was started on a venetoclax‐based regimen with cyclophosphamide, vincristine, dexamethasone alternating with methotrexate and cytarabine (mini‐hyper‐CVD) with venetoclax [10, 11, 12]. Given the lack of standard approaches to her unique condition, the patient was counseled heavily on the risks and benefits of this novel approach, and appropriate informed consent was obtained. After Cycle 1A of treatment, BM biopsy showed 80% hypercellularity with 2%–5% involvement of B‐ALL and 10%–15% involvement of T‐PLL. She completed Cycle 1B with recovery marrow showing relapsed B‐ALL—80%–90% hypercellularity with 90% involvement of B‐ALL and 5% involvement of T‐PLL. FISH panel showed the same rearrangements as initial diagnostic BM biopsy, with the addition of 19p13.3 (*TCF3*) loss. NGS from this BM showed *KMT2A (MLL)::MLLT1* fusion, *DDX3X* T275 M (VAF 1.5%), *JAK3* M511I (VAF 7.9%), and *ATM* K2811del (VAF 8.4%).

Due to refractory B‐ALL, she was escalated to HyperCVAD without venetoclax. Her B‐ALL unfortunately did not respond after the first Cycle 1A with BM showing 90% cellularity with 70%–80% involvement of B‐ALL and 10%–15% T‐PLL. Given concerns that B‐ALL was the more aggressive of the two malignancies and should be preferentially targeted, the patient was then started on single‐agent inotuzumab. After one cycle of inotuzumab (3 weeks), BM biopsy showed 60% cellularity with 30% involvement of B‐ALL and 60% involvement of T‐PLL. One week after BM biopsy, she had an abrupt increase in WBC count from 33 × 10^9^/L to 210 × 10^9^/L, with PB flow cytometry showing 86% of cells with B‐ALL phenotype and 5.9% of cells with T‐PLL phenotype. After debulking of WBC to 20 × 10^9^/L with dexamethasone, hydroxyurea, and vincristine, she started blinatumomab 9 mcg daily with titration to 28 mcg daily. This was combined with venetoclax 100 mg daily, dose‐reduced as she was on posaconazole [13]. Her PB flow cytometry at the end of her first cycle of blinatumomab showed 65% population of B lymphoblasts and 26% T‐PLL. Unfortunately, she had an extended hospitalization that was complicated by invasive pulmonary aspergillosis, and she was not a candidate for further treatment. She was discharged with hospice care and succumbed to her illness 16 months after initial diagnosis of T‐PLL and 7 months after initial diagnosis of B‐ALL.

## 3 Discussion

To our knowledge, this is the first case in the literature of a patient with T‐PLL followed by B‐ALL. She was initially classified at PTCL, not otherwise specified, and treated with CHOEP prior to re‐evaluation, finding a *TCL1* rearrangement and diagnosing T‐PLL. Due to progressive cytopenias, patient started T‐PLL‐directed treatment with intravenous alemtuzumab, with eventual addition of pentostatin due to persistent leukocytosis. Surprisingly, a repeat PB flow cytometry after an inadequate response revealed a new neoplastic population of B‐lymphoblasts diagnostic of B‐ALL. Initially, a treatment approach using mini‐hyper‐CVD with venetoclax was used to combat both diseases. This regimen has previously been shown to be safe and effective in early phase studies of patients with heavily treated B‐ALL [10]. T‐PLL has demonstrated sensitivity to venetoclax in both laboratory and pre‐clinical studies, presumably due to high levels of BCL‐2 expression in T‐PLL [11]. Due to refractory disease, her treatment was soon escalated to HyperCVAD to focus on the more aggressive B‐ALL. She was refractory to HyperCVAD as well and then started single‐agent inotuzumab with initial response in B‐ALL; however, her T‐PLL clone started rising, followed by her B‐ALL clone. Treatment with blinatumomab and venetoclax was attempted to control both neoplastic populations, but she had persistent disease and ultimately developed an invasive fungal infection from prolonged cytopenias. Of note, blinatumomab, a bispecific T‐cell engager antibody, was reserved for a later line of therapy because of the unknown role the aberrant T‐PLL population would play in its efficacy or toxicity. With T‐PLL comprising a significant portion of total lymphocytes, it was unclear if a T‐cell‐engaging therapy would be effective. Furthermore, there were concerns about T‐cell depletion from upfront alemtuzumab. In addition, the patient in our case did not have access to transplant or CAR‐T cells due to financial constraints, nor was she ever stable to receive either treatment option.

Given the persistent presence of a *JAK3* mutation, ruxolitinib in combination with venetoclax was considered a potential treatment plan, as early data suggest safety and efficacy in T‐PLL and acute myeloid leukemia [14, 15]. Given the aggressive nature of B‐ALL, the patient's good performance status, and uncertainty over the role of *JAK3* as a driver in the B‐ALL clone, we opted for a traditional chemotherapy‐based approach. After the failure of HyperCVAD with venetoclax, ruxolitinib was again discussed, especially given the above concerns with T‐cell‐engaging therapies. However, given that venetoclax was ineffective with chemotherapy, single‐agent ruxolitinib, with a lack of precedent in B‐ALL, was felt to be an inadequate treatment. Ruxolitinib may have been a viable option had there been opportunity for an additional line of treatment.

**FIGURE 2:**
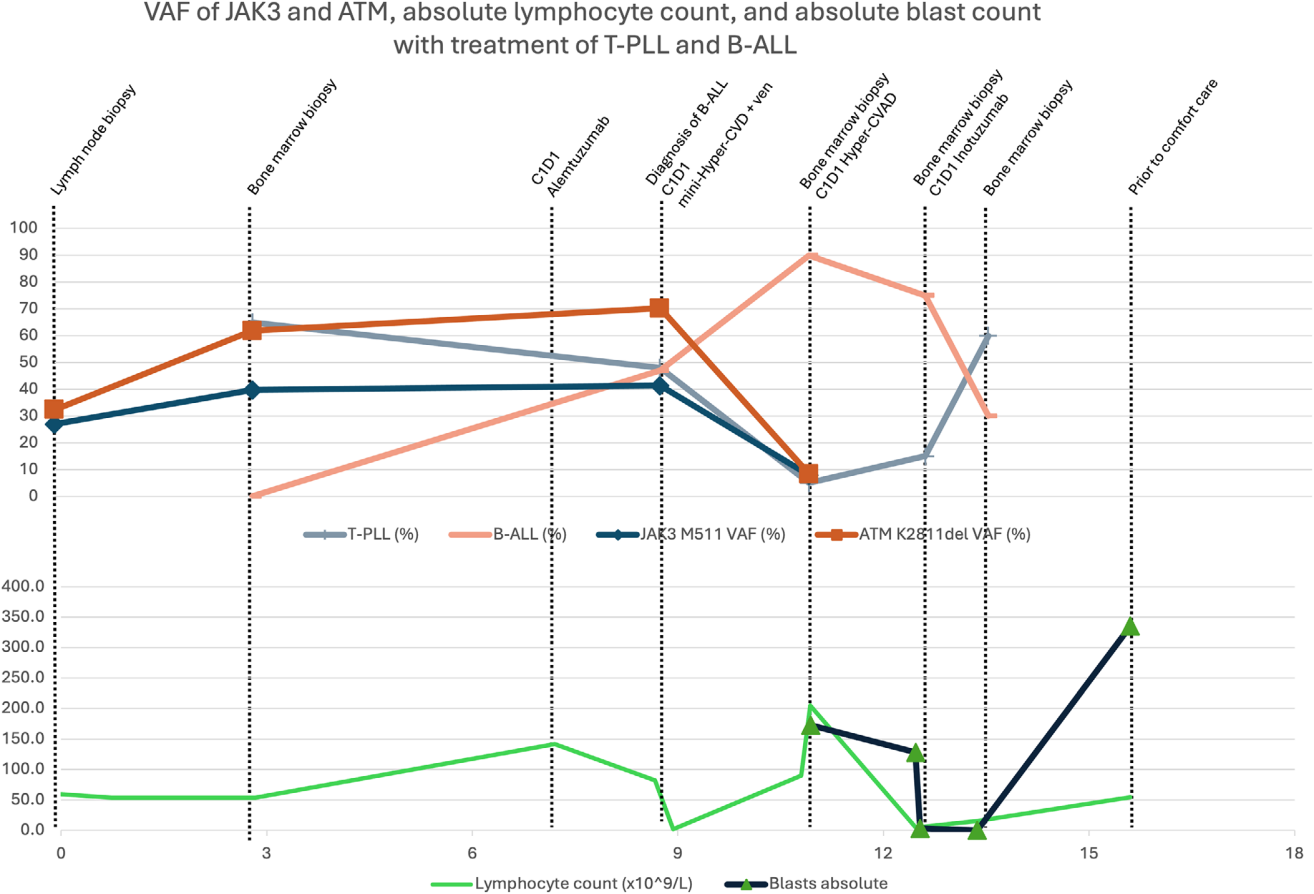
Variant allele frequency (VAF) of JAK3 M511 and ATM K2811del obtained via FoundationOne next‐generation sequencing (NGS), trended alongside populations of T‐prolymphocytic leukemia (T‐PLL), B‐cell acute lymphoblastic leukemia (B‐ALL), absolute lymphocyte and blast counts while undergoing treatment. Month 3 is the initial yield of T‐PLL in bone marrow after having received 3 cycles of CHOEP. Month 9 represents initial diagnosis of B‐ALL; however, NGS studies are run on peripheral blood due to dry aspirate. Month 11 shows refractory B‐ALL in bone marrow after Cycle 1B mini‐hyper CVD with venetoclax.

It is conceivable that the T‐PLL clone and the B‐ALL clone may have risen from a common *JAK3‐*mutated ancestral progenitor. Similarly, a germline condition may have predisposed the patient to both malignancies. Her *JAK3* VAF remained high when she was treated for T‐PLL with alemtuzumab and pentostatin and thereafter diagnosed with B‐ALL. VAF of *JAK3* M511I in BM was 39.7% at initial T‐PLL diagnosis and 41.3% on PB on her B‐ALL diagnosis. The VAF of *JAK3* M511I and *ATM* K2811del was substantially lower (at ∼8%) when the BM was involved by a low level of T‐PLL (∼5% of BM) and a high level of B‐ALL (90% of BM) (Table 1, Figure 2). Acquisitions of a *TCL1* rearrangement and *ATM* mutation may have driven the T‐PLL, and the development of the *KMT2A* fusion may have driven the B‐ALL.

**TABLE 1:** Next‐generation sequencing and variant allele frequency (VAF) of mutations after initial biopsy of submental lymph node showing a T‐cell lymphoma. B‐ALL was diagnosed 6 months after the initial bone marrow diagnosis of T‐PLL, after receiving 7 weeks of alemtuzumab and three weeks of pentostatin after a period of observation. Bone marrow procedure for the initial diagnosis of B‐ALL did not yield an aspirate, and studies were collected on peripheral blood.

**Source**	**Time (months)**	**Results**	**Alteration**	**VAF**
Bone marrow	3	Hypercellular, 70%–80% 60%–70% T‐PLL	*JAK3* M511I NM_000215.3:c.1533 G>A (p.MF11I)	39.7%
			*ATM* K2811del NM_000051.3:c.8430_8432del (p.K2811del)	61.8%
Bone marrow	9	Hypercellular, 100% 90% B‐ALL 10% T‐PLL		
Peripheral blood	9	47% B‐ALL 48% T‐PLL	*KMT2A::MLL1*	
			*JAK3* M511I NM_000215.3:c.1533 G>A (p.MF11I)	41.3%
			*ATM* K2811del NM_000051.3:c.8430_8432del (p.K2811del)	70.2%
			*RB1* rearrangement exon 4	
Bone marrow	11	Hypercellular, 80%–90% 90% B‐ALL	*KMT2A::MLL1*	
			*JAK3* M511I NM_000215.3:c.1533 G>A (p.MF11I)	7.9%
		5% T‐PLL	*ATM* K2811del NM_000051.3:c.8430_8432del (p.K2811del)	8.4%
			*DDX3X* T275 M NM_001356.3:c.824C>T (p.T275 M)	1.5%

The constellation of NGS results may suggest an alternative model of pathogenesis. T‐PLL and B‐ALL could be derived from a common stem cell progenitor, with acquisition of *JAK3, ATM*, and *TCL1* rearrangement leading to T‐PLL, while acquisition of *KMT2A (MLL)::MLLT1* fusion leading to B‐ALL, the latter representing t‐BALL. The patient did receive the topoisomerase II inhibitor etoposide upon her initial diagnosis of PTCL, and subsequently developed a *KMT2A* rearrangement, raising the possibility of t‐BALL; however, she received only a truncated course of etoposide (900 mg/m2 total through three cycles), and she only had a latency period of 8 months after exposure. Nevertheless, the possibility of t‐BALL remains.

## 4 Conclusion

This report highlights the diagnosis and management options of a previously undescribed hematologic disorder, T‐PLL with subsequent concurrent B‐ALL. This case underscores the importance of continual re‐evaluation if a patient is refractory to standard treatments. A limitation is that this is one case at a single center at a safety‐net hospital. In addition, direct comparisons cannot be made between VAF between relapses, as BM biopsy on diagnosis of B‐ALL did not result in any aspirate for testing, and therefore, studies had to be performed on PB. Ultimately, the biggest challenge in the management of this case was finding a regimen that would successfully treat both malignancies, which we were unfortunately unable to do. However, understanding the pathogenesis may provide insight into future treatment options as more targeted therapies are developed.

## Author Contributions

V.M.P. made substantial contributions to the conception, design, analysis of this work, figure creation, drafted this work, and made final edits to the documentation based on co‐author feedback. J.H. contributed to the conception and analysis of the work, made significant contributions to figures, and reviewed documentation for final edits. S.L. contributed to data collection, formal analysis, and figure creation. M.C. critically reviewed the methodology of the work and contributed to the final manuscript creation. P.P. contributed to the conception and analysis of the work and reviewed the documentation for final edits. W.C. made significant contributions to the conception, data analysis, and creation of figures, and contributed to writing and editing. R.K. likewise made significant contributions to the conception and data analysis, as well as contributed to the writing and editing of this product. All authors approved the final version of this work to be published. They take responsibility for the integrity of the work.

## Funding

The authors have nothing to report.

## Ethics Statement

The authors have nothing to report.

## Conflicts of Interest

The authors declare no conflicts of interest.

## Data Availability

The data that support the findings of this case report are not publicly available due to privacy reasons, but are available from the corresponding author upon reasonable request.
